# Growth Hormone Ameliorates the Radiotherapy-Induced Ovarian Follicular Loss in Rats: Impact on Oxidative Stress, Apoptosis and IGF-1/IGF-1R Axis

**DOI:** 10.1371/journal.pone.0140055

**Published:** 2015-10-14

**Authors:** Yasmen F. Mahran, Ebtehal El-Demerdash, Ahmed S. Nada, Reem N. El-Naga, Azza A. Ali, Ashraf B. Abdel-Naim

**Affiliations:** 1 Department of Pharmacology & Toxicology, Faculty of Pharmacy, Ain Shams University, Cairo, Egypt 11566; 2 National Center for Radiation Research and Technology (NCRRT), Atomic Energy Authority, Cairo, Egypt 113701; 3 Department of Pharmacology & Toxicology, Faulty of Pharmacy, Al-Azhar University, Cairo, Egypt 11787; University of Kansas Medical Center, UNITED STATES

## Abstract

Radiotherapy is one of the standard cytotoxic therapies for cancer. However, it has a profound impact on ovarian function leading to premature ovarian failure and infertility. Since none of the currently available methods for fertility preservation guarantees future fertility, the need for an effective radioprotective agent is highly intensified. The present study investigated the mechanisms of the potential radioprotective effect of growth hormone (GH) on γ irradiation-induced ovarian failure and the impact of the insulin like growth factor 1 (IGF-1) in the underlying protection. Immature female Sprague-Dawley rats were either exposed to single whole body irradiation (3.2 Gy) and/or treated with GH (1 mg/kg s.c). Experimental γ-irradiation produced an array of ovarian dysfunction that was evident by assessment of hormonal changes, follicular development, proliferation marker (PCNA), oxidative stress as well as apoptotic markers. In addition, IGF-1/IGF-1R axis expression was assessed using real-time PCR and immunolocalization techniques. Furthermore, after full maturity, fertility assessment was performed. GH significantly enhanced follicular development and restored anti-Mullerian hormone serum level as compared with the irradiated group. In addition, GH significantly ameliorated the deleterious effects of irradiation on oxidative status, PCNA and apoptosis. Interestingly, GH was shown to enhance the ovarian IGF-1 at transcription and translation levels, a property that contributes significantly to its radioprotective effect. Finally, GH regained the fertility that was lost following irradiation. In conclusion, GH showed a radioprotective effect and rescued the ovarian reserve through increasing local IGF-1 level and counteracting the oxidative stress-mediated apoptosis.

## Introduction

Radiotherapy is one of the most effective cytotoxic therapies for cancer [1]. About eighty percent of cancer patients need radiotherapy at some time or other [2]. Unfortunately, normal tissues adjacent to the tumor are unavoidably exposed to radiation and reduce its therapeutic gain [1]. However, physicians viewed these drastic effects as an acceptable cost of curative radiotherapy [3, 4]. While the damage induced is reversible in some tissues, it is progressive and permanent in the ovary [5]. The ovaries are exposed to significant doses of irradiation when radiotherapy is used to treat pelvic and abdominal diseases or through the whole body irradiation [6]. In this context, radiation to the brain can damage the pituitary gland, leading to deregulation of the two gonadotropins [follicle stimulating hormone (FSH) and luteinizing hormone (LH)] required to maintain normal ovarian function [7].

In addition, radiation directly destroys ovarian follicles leading to depletion of the primordial pool [8] and consequently, predisposes women to premature menopause and infertility [9, 10], during or shortly after completion of irradiation [5]. Premature ovarian failure (POF), which is a common cause of infertility in premenopausal women, defined as cessation of ovarian function with elevated gonadotropins and low estradiol (E2) serum levels before the age of 40. In most cases, loss of fertility is either due to the absence of follicles or the inability of the remaining follicles to respond to stimulation [11]. In ovarian failure, ovarian follicles do not respond to a high level of FSH induced by radiation and do not secrete estradiol (E2) and consequently, ovarian follicles are driven to apoptosis [12]. Indeed, apoptosis was identified as the mechanism responsible for oocyte loss induced by anti-cancer therapy [13].

Some strategies have been trying to reduce the gonadotoxic effect of radiotherapy. However, very mixed results have been obtained and none of the currently available methods for ovarian protection is ideal or guarantees future fertility [14]. Thus, the need for more effective strategy to protect the ovary from the cytotoxicity of radiotherapy is highly intensified, not only to maintain the oocyte quality, but also, to preserve hormone production, which supports fertility.

Growth hormone (GH) is an anabolic hormone with pleiotropic effects on growth, differentiation and metabolism of cells. Recently, hormones of the somatotrophic axis were shown to play regulatory roles in reproduction. GH action is mediated through enhancing serum and local (ovarian) IGF-1 levels and through direct GH receptor (GHR)–mediated effects in the ovary [15]. Also, GH/ IGF-1 axis was reported to increase the sensitivity of ovaries to gonadotropins stimulation and thus, enhance steroidogenesis and follicular development [16]. In addition, many women with GH deficiency suffer a condition of subfertility and require assisted reproductive technologies to conceive [17, 18]. Clinically, poor responders with hypogonadotropic hypogonadism, who were co-stimulated with GH, achieved more oocytes, higher fertilization and pregnancy rates [19, 20]. It was reported that the use of GH reduced the human menopausal gonadotropin dose and duration required for ovulation induction and improved success rates [21].

Moreover, human GH (hGH) administration had a radioprotective effect both *in vivo* and *in vitro* possibly due to DNA repairing mechanisms [22, 23]. **Baeza and coworkers** [24] have recently found out that hGH administration or IGF-1 overexpression reduces the mitochondria-mediated oxidative stress and induces the anti-oxidant systems in the liver and kidney of ovariectomized rats. Accordingly, we suggested that GH could participate in radiotherapy-induced gonadotoxicity through its anti-oxidant effect.

Nevertheless, little information was found on the specific molecular pathways implicated in GH effects in the ovary [25] and no data has been reported on the radioprotective role of GH in ovarian damage induced by γ-irradiation *in vivo*. Therefore, the present study was designed to explore the modulatory effects of GH on radiation-induced ovarian dysfunction *in vivo* as well as the possible underlying mechanisms; particularly, its impact on the ovarian reserve, oxidative stress, proliferation, apoptosis and the impact on IGF-1/IGF-1R axis.

## Material and Methods

### 2.1. Drugs and chemicals

Recombinant human growth hormone (Genotropin, somatropin 1.3 mg/4iU inj.) was obtained from Pharmacia & Upjohn Co, Div of Pfizer Inc. (NY). Bovine serum albumin was obtained from Sigma Chemical Co. (St Louis, Missouri). Dipotassium hydrogen phosphate (K2HPO4) and potassium dihydrogen phosphate (KH2PO4) were purchased from El-Nasr Chemical Co (Egypt). Glutathione peroxidase and reductase assay kit were purchased from Randox Laboratories (Antrim, UK) and Trevigen, Inc. (Gaithersburg, Maryland), respectively. Mag-NA Pure Compact RNA Isolation Kit and LightCycler_RNA Master SYBR Green I were obtained from Roche Diagnostics, (Indianapolis, Indiana)/Roche Applied Science (Mannheim, Germany). Beta-actin primer sequence design was carried out according to **Peinnequin and coworkers** [26], while other primer sequences were designed according to **Loh and his colleagues** [27]. All other chemicals and solvents were of highest-grade commercial available.

### 2.2. Gamma irradiation

The animals were whole-body irradiated using a Cesium (^137^CS) source, Gamma Cell-40 biological irradiator, at the National Centre for Radiation Research and Technology (NCRRT), Cairo, Egypt. Animals were exposed to a single dose of irradiation (3.2 Gy) with a standardized dose rate of 0.48 Gy/min. This dose represents the LD_20_ according to the study of **Lee and coworkers** [28]. The plastic boxes containing rats were positioned in a chamber fixed to the irradiation equipment.

### 2.3. Animals

The study was conducted according to ethical guidelines of Faculty of Pharmacy, Ain Shams University, Egypt. Immature female Sprague- Dawley rats (23 days of age) were obtained from the Nile Co. for Pharmaceuticals and Chemical industries, Cairo, Egypt. Immature female rats were chosen at this age (23 days), according to previous studies [29, 30]. At this juvenile stage just before puberty, immature rats were used in order to investigate the full primordial stock, the major determinant of reproductive lifespan and the most sensitive follicle type for radiation.

Rats were acclimated for one week before experimentation and were housed in an air-conditioned atmosphere, at a temperature of 25°C with alternatively 12 hour light and dark cycles. They were kept on a standard diet and water ad libitum. Standard diet pellets (El-Nasr, Abu Zaabal, Egypt) contained not less than 20% protein, 5% fiber, 3.5% fat, 6.5% ash and a vitamin mixture.

### 2.4. Experimental design

**Fig 1:**
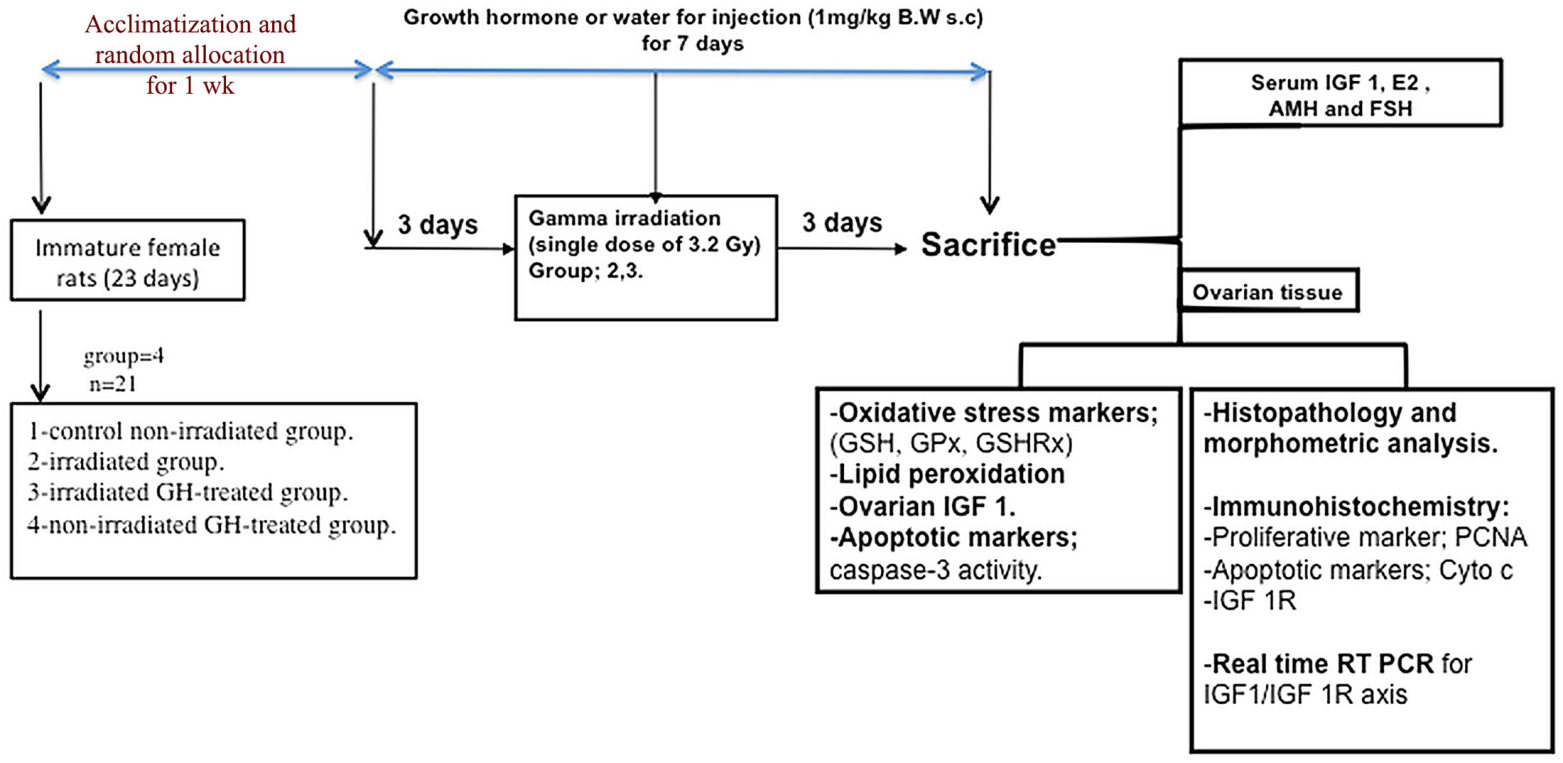
Schematic diagram of the experimental design.

Immature rats were randomly assigned to four groups (twenty one rats per group) and treated for one week as follows; 1) control group: non-irradiated rats received water for injection (0.5 ml/100 g B.W s.c.) once daily; 2) irradiated rats; received water for injection (0.5 ml/100 g B.W s.c.) once daily; 3) irradiated GH-treated rats received rhGH in water for injection (1 mg/kg B.W s.c.) once daily and 4) non-irradiated GH-treated rats received rhGH in water for injection (1 mg/kg B.W. s.c.) once daily. Rats were injected s.c. with either water for injection or GH for 7 days starting 3 days before irradiation and lasting 3 days post-irradiation as shown by a schematic diagram (Fig 1). In order to determine which dose of GH is more appropriate as radioprotective; two doses of GH (0.5 and 1 mg/kg) were used for hormonal assessment and depending on the results obtained from this experiment, the appropriate GH dose was chosen and used for further mechanistic studies. However, a previous study conducted by **Gómez-de-Segura et al.** [22] used rhGH at a dose of 1mg/kg as a radioprotective. Animal body weights were recorded daily till the sacrifice day. Four days post-irradiation; five rats per each group were utilized for histopathology and immunohistochemistry studies, six rats per each group were utilized for biological assessment and ten rats per each group were utilized for fertility assessment. Blood samples were collected from the retro-orbital plexus and allowed to clot. Afterwards, rats were sacrificed; ovarian tissues were dissected, washed with ice-cold saline and then weighed.

### 2.5. Tissue collection and processing

Serum was separated by centrifugation at 3000 g for 15 min and kept frozen at– 80°C until used for the assessment of Anti-mullerian hormone (AMH), 17 β-estradiol (E2), FSH and IGF-1. Ovarian samples were homogenized in 1:10 (w:v) saline (PH 7.4) or according to the available KIT lysis buffer with an Ultra Turrax homogenizer. Afterwards, the supernatant was obtained by centrifugation at 10,000 x*g* for 15 minutes and kept at –80°C until assessment of oxidative stress markers; reduced glutathione (GSH) level, glutathione peroxidase (GPx), glutathione reductase (GSHRx) activities and lipid peroxidation level. Apoptotic markers (caspases-3 activity) and the local IGF-1 assay were also assessed. In addition, five ovaries from each group were fixed in appropriate buffer for the histopathological examination and morphometric analysis as well as immunohistochemical detection of proliferating marker; proliferating cell nuclear antigen (PCNA), apoptotic marker; cytochrome C (Cyto c) as well as growth factor receptor; IGF-1 R. Furthermore, ovarian tissue samples were kept frozen at –80°C until processed for RNA extraction and amplification of IGF-1 and IGF-1R (Fig 1).

### 2.6. Assessment of circulating levels of AMH, E2 and FSH

Assessment of the follicular reserve was done by measurement of serum AMH using AMH ELISA kits (Uscn Life Science Inc. Wuhan). Also, serum E2 and FSH were measured using ELISA kit (EIAAB, Inc., Wuhan) for estimating the circulating levels of serum E2 and the commercially available radioimmunoassay Kit (rat FSH IRMA C.T., IBL International GMBH, Germany) was used to estimate the serum FSH levels. The intra- and inter-assay coefficients of variation were found to be as follows; less than 2.8% and 12% respectively for AMH, less than 4% and 11.6%, respectively for E2, and less than 3% and 11%, respectively for FSH. The minimum detectable concentrations for AMH, E2 and FSH were 0.078 ng/ml, 3.6 pg/ml and 0.2 ng/ml, respectively.

### 2.7. Assessment of serum and local ovarian IGF-1 levels

Serum and ovarian IGF-1 were assayed using the commercially available kits; Quantikine rat/mouse IGF-1 immunoassay kit (R&D Systems, Inc., Minneapolis, Minnesota). The intensity of the yellow color measured at 450 nm was in proportion to IGF-1 levels. Serum and local IGF-1 were expressed as ng/ml and ng/mg protein respectively. Protein was measured according to the method of **Lowry** [31] using bovine serum albumin as standard.

### 2.8. Assessment of oxidative stress markers

GSH and lipid peroxidation were determined in ovarian homogenate using the commercially available assay kit; GSH assay Kit from Sigma-Aldrich (St Louis, Missouri) and OxiSelect™ TBARS assay Kit (Cell Biolabs, Inc.), respectively. According to the manufacturer's instructions, ovarian GSH content was assayed based on **Fernandez-Checa and Kaplowitz** method [32]. In addition, Lipid peroxidation was determined by estimating the level of thiobarbituric acid reactive substances (TBARS), measured as malondialdehyde (MDA) content, according to the method of **Mihara and Uchiyama** [33]. Quantities of ovarian GSH and MDA were expressed as μmol and nmol per gm wet tissue, respectively.

GPx activities were determined spectrophotometrically based on **Paglia and Valentine method** [34]. GPx catalyses the oxidation of GSH by Cumene Hydroperoxide; in the presence of GSHRx and NADPH, the oxidized glutathione (GSSG) is immediately converted to the reduced form (GSH) with a concomitant oxidation of NADPH to NADP^+^. The rate of decrease in absorbance at 340 nm was calculated and specific activities were expressed as a unit/ mg protein. As well, GSHRx activity was measured spectrophotometrically according to the method of **Carlberg and Mannervik** [35]. GSHRx catalyzes GSSG reduction to 2 GSH along with oxidation of NADPH to NADP+. This is accompanied by a decrease in absorbance at 340 nm and is directly proportional to the GSHRx activity in the sample. The rate of decrease in absorbance at 340 nm per minute was calculated and GSHRx activity was expressed as mU/ mg protein. Notably, protein was determined according to the method of **Lowry** [31] using bovine serum albumin as standard.

### 2.9. Assessment of apoptotic markers

Immunohistochemical detection of Cyto c was carried out. In addition, ovarian caspase-3 activity was assessed using caspase-3 colorimetric assay Kit (R & D systems, Inc.) according to the manufacturer's instructions. Briefly, this assay quantifies caspase-3 activation by measuring the cleavage of caspase-3 substrate; DEVD-pNA releasing the chromophore pNA, which can be measured using a microplate reader at a wavelength of 405 nm. The level of caspases-3 enzymatic activity was directly proportional to the color reaction and was expressed as nmol/mg protein. Protein was determined according to **Lowry method** [31].

### 2.10. Histopathological examination

Ovaries and uterine tissues were fixed in 10% formalin overnight and embedded in paraffin. Serial sections of 4 μm thick were stained with hematoxylin and eosin for light microscopic histological examination. In all ovarian samples, at least three ovarian sections, after the fifth cut, were chosen to count the number of follicles and to evaluate follicular development throughout the entire ovary using a digital video camera mounted on a light microscope (CX21, OLYMPUS, JAPAN). Follicles were classified depending on their follicular development. Follicles were classified as primordial if they contained an oocyte surrounded by a single layer of spindle-shaped granulosa cells. Follicles were classified as pre-antral if they contained an oocyte with a visible nucleolus, more than one layer and less than five layers of granulosa cells and lacked an antral space. Follicles were classified as antral if they contained an oocyte with a visible nucleolus, more than five layers of granulosa cells and/ or an antral space as described previously [36]. Atretic follicles were identified due to the presence of a degenerating oocyte or pyknotic granulosa cells [37]. The diameters of all antral follicles in the sections were measured in two perpendicular axes according to **Benedict and coworkers** [38] using image analysis software (Image J, 1.46a, NIH, USA). The mean of the two diameters was calculated to obtain the mean diameter per follicle. The data are expressed as mean ± SEM.

### 2.11. Immunohistochemistry

#### 2.11.1. Proliferative marker

Immunohistochemical analysis of PCNA [39] was carried out to assess the proliferative capacity of granulosa cells in all groups. Paraffin embedded ovarian sections of 3 μm thick were rehydrated first in xylene and next in graded ethanol solutions. Then, the slides were blocked with 5% bovine serum albumin in Tris buffered saline (TBS) for 2 h. Immunohistochemical analyses were performed using the standard streptavidin-biotin-peroxidase procedure. The sections were incubated with a mouse anti-PCNA monoclonal antibody (Thermo Fisher Scientific, Pittsburgh, Pennsylvania; Cat. No. MS-106-R7) overnight at 4°C. After rinsing thoroughly with TBS, the sections were incubated with a biotinylated goat anti-rabbit secondary antibody for 10–15 min, then the horseradish-peroxidase-conjugated streptavidin solution was added and incubated at room temperature for 10–15 min. Sections were then washed with TBS and incubated for 5–10 min in a solution of 0.02% diaminobenzidine containing 0.01% H_2_O_2_. Counter staining was performed using hematoxylin, and the slides were visualized under a light microscope. Positive staining was defined as intense reddish-brown staining of the nucleus and negative was defined as light or diffuse staining of the nucleus and cytoplasm [40]. To obtain an estimate of the percentage of proliferating cells, the percentage of nuclei positively stained for PCNA from the total number of granulosa cells was estimated in six high-power fields (40×) using a digital video camera.

#### 2.11.2. Apoptotic markers

As mentioned before, ovarian sections were incubated with primary antibody, a mouse anti-cytochrome c monoclonal antibody (Thermo Fisher Scientific, Cat. No. MS-1192-R7). In addition, a biotinylated goat anti-rabbit was used as secondary antibody. Fractions of ovarian Cyto c DAB-positive immunoreactive areas were calculated automatically in six high-power fields (20×) and the percentage of immunopositive cells to the total area of the microscopic field was calculated using a digital video camera mounted on a light microscope (CX21, OLYMPUS, JAPAN). All steps for immunohistochemical evaluation were carried out using image analysis software (Image J, 1.46a, NIH, USA).

#### 2.11.3. Insulin like growth factor one receptor expression

Immunohistochemical localization of IGF-1R protein was done according to the method previously mentioned. A rabbit Anti-IGF 1R polycolonal antibody (Novus biological Inc. Littleton, Colorado) was used as primary antibody and we used a biotinylated goat anti-rabbit, as secondary antibody. IGF-1R staining intensities were calculated as the mean optical densities (OD) of selected areas in six high-power fields (20×) using the automated image analysis software (Image J, 1.46a, NIH, USA) as previously described [41, 42], and results were expressed as the mean OD.

### 2.12. RNA extraction and amplification using Real Time RT PCR for IGF-1 and IGF-1R

Total RNA was extracted from the ovaries of the rats in different groups using the MagNA Pure Compact RNA Isolation Kit (Roche Diagnostics, Roche Applied Science, Germany) and the MagNA Pure Compact Instrument (Roche Applied Science, USA) according to the manufactures instructions. Ovarian tissues (10 mg) were homogenized and pre-lysed by incubation with the Lysis/Binding Buffer containing a chaotropic salt and Proteinase K and the Instrument was used to complete the software- guided RNA isolation steps automatically.

cDNA synthesis (RT step) and the subsequent Real-time quantitative PCR were carried out using a one-step LightCycler_RNA Master SYBR Green I kit (Roche Diagnostics, Roche Applied Science, Germany) and a LightCycler rapid thermal cycler system (Roche Diagnostics Ltd., Lewes, UK) according to the manufacturer’s instructions. The primers for the PCR reactions were as follows: rat β-actin, forward, 5′-GTAAAGACCTCTATGCCAACA-3′, reverse, 5′-GGACTCATCGTACTCCTGCT-3′ (Annealing temperature 62°); IGF-1, forward, 5′-AAAATCAGCAGTCTTCCAAC-3′, reverse, 5′-AGATCACAGCTCCGGAAGCA-3′ (Annealing temperature 62°C); IGF-1R, forward,5′-TCCACCATAGACTGGTCTCT-3′,reverse,5′-ACGAAGCCATCTGAGTCA CT-3′ (Annealing temperature 56°C). A typical protocol included a 60 s denaturation step followed by 45 cycles with a 7 s denaturation step at 95°C, a 10 s annealing step and a 15 s extension step at specified temperature for each primer. Relative quantification ratio of gene expression was done according to **Pfaffl** [43] by the aid of relative expression software tool multiple condition solvers (REST-MCS_) version 2, 2006 using the following equation:

$${\text{Ratio }=\text{ E}}^{\Delta \text{CT }\left(\text{control}-\text{experiment}\right)\text{ target}}{/\text{E}}^{\Delta \text{CT }\left(\text{control}-\text{experiment}\right)\text{ ref}.}$$

Where E is the PCR amplification efficiency, ΔC_T_ the difference in the crossing points.

### 2.13. Fertility assessment

All groups were tested daily for estrous cycles using vaginal lavage techniques. Vaginal lavage was performed in the morning by flushing the vagina with 20 μl of distilled water and subsequently aspirated, then smeared onto a glass slide. The flushed vaginal fluid was fixed with 70% ethanol and examined microscopically using methylene blue stain. Female rats from all groups (n = 10 in each group) were mated with age related, and sexually experienced males at about 60-day post natal (dpn) for up to 3 sexual cycles (3 estrus). One male was housed with a group of 2 or 3 females. Vaginal swabs were used to ensure the presence of sperms to assess mating. Pregnant females at 14–15 d of gestation were isolated. Number of pregnant females and newborn pups were counted and kept with their mother until 2 dpn to check breastfeeding and eventual lethality.

### 2.14. Statistical analysis

Data were presented as mean ± SEM. Multiple comparisons were performed using ANOVA followed by Tukey–Kramer as a post-hoc test. The difference in number of pups was compared using Kruskal-Wallis's test followed by Dunn's multiple comparisons as a post-hoc test. The 0.05 level of probability was used as the criterion for significance. All statistical analyses were performed using Instat version 3 software package. Graphs were sketched using GraphPad Prism (ISI^®^ software, San Diego, USA) version 5 software.

## Results

### 3.1. Body weight and ovarian weight changes

Four days after irradiation, rats gained less weight and their ovaries weighed less than the control group by about 0.4 and 0.52 fold respectively as shown in Table 1. In contrast, irradiated GH-treated rats gained more weight when compared with the irradiated group. In addition, their ovarian weights were significantly higher than irradiated group. However, they were still lower than the control ones. The GH-treated rats showed non-significant changes to body weight gain and ovarian weights when compared with the control group. The relative ovary weight in the irradiated group significantly was lowered by 0.48 fold as compared to control group but concurrent GH treatment could not change this reduction (Table 1).

**Table 1:** Effect of Growth hormone (1mg/kg s.c.; once daily for 1 wk) on body and ovarian weight and the relative ovary weight in rats subjected to single dose whole-body irradiation (3.2 Gy).

Treated groups	Body wt differences (g)	Ovarian weight (mg)	Relative ovary wt (mg/100g body weight)
**Control**	**17.90 ± 1.14**	**42.20 ± 2.20**	**75.04 ± 5.3**
**IR**	**7.30 ± 0.58** ^**a**^	**20.80 ± 0.48** ^**a**^	**36.16 ±1.00** ^**a**^
**IR/GH**	**16.41 ± 0.99** ^**b**^	**30.73 ± 3.25** ^**a,b**^	**51.24 ±6.35** ^**b**^
**GH**	**20.12 ± 1.07 b**	**32.33 ± 0.33** ^**a,b**^	**47.00±0.86 a**

-Data expressed as mean ± SEM (n = 10).

-a or b: Significantly different from control or radiation group, respectively at p <0.05 using one-way ANOVA followed by Tukey–Kramer as a post-hoc test.

IR: irradiation; IR/GH: irradiation/Growth hormone; GH: Growth hormone.

### 3.2. Serum hormone levels

AMH has recently received much attention as a marker of ovarian reserve. As shown in Table 2, the irradiated rats had undetectable levels of serum AMH and E2 reaching 0.11 and 0.18-fold of the control values respectively. In the irradiated GH-treated rats, AMH levels were nearly normalized and serum E2 levels significantly increased by 2.6 fold as compared to the irradiated group. However, they were still lower than that of the control group. In addition, GH (1mg/kg) alone for one week showed no changes in serum AMH or E2 levels when compared with the control values. Furthermore, a marked increase in serum FSH level was found four days post-irradiation reaching 4.7 fold as compared with the control group. This defect was significantly resolved in the irradiated group treated with GH (1mg/kg) (Table 2). However, GH alone showed a modest but significant increase in FSH level when compared to the control group. The irradiated rats treated with GH (0.5 mg/kg) did not show any significant difference in hormonal levels when compared with the irradiated group (Table 2). Accordingly, 1mg/kg rhGH was chosen for the further protective mechanistic studies.

**Table 2:** Effect of Growth hormone (0.5, 1mg/kg s.c. once daily for 1 wk) on serum hormone levels in rats subjected to single dose whole-body irradiation (3.2 Gy).

Treated group	AMH* (ng/ml)	FSH* (ng/ml)	E2* (pg/ml)
**Control**	**5.41 ± 0.504**	**22.76 ± 1.646**	**29.60 ± 1.905**
**IR**	**0.61 ± 0.105** ^**a**^	**108.7 ± 2.158** ^**a**^	**5.28 ± 0.216** ^**a**^
**IR/GH 1mg**	**6.7 ± 0.40** ^**b**^	**87.02 ± 1.76** ^**a,b**^	**14.18 ± 0.50** ^**a,b**^
**IR/GH 0.5 mg**	**0.713 ± 0.03** ^**a**^	**101.66 ± 1.90** ^**a**^	**8.00 ± 0.60** ^**a**^
**GH**	**5.7 ± 0.033** ^**b**^	**44.70 ± 5.91** ^**a,b**^	**24.30 ± 2.67** ^**b**^

-Data expressed as Mean ± SEM (n = 6).

-a, b: Significantly different from control, radiation, respectively at p <0.05 using one-way ANOVA followed by Tukey–Kramer as a post-hoc test.

*AMH: Anti-mullerian hormone; FSH: follicle stimulating hormone; E2: estradiol.

### 3.3. Folliculogenesis and proliferation markers

AMH level has been reported to be strongly correlated with the antral follicle count (AFC), another marker of ovarian reserve [44]. Therefore, morphometric analysis of follicles types and numbers was carried out and results demonstrated that the irradiated ovaries displayed a sequential decrease in the number of all follicular classes. The primordial pool showed a significant decrease to about 0.15 fold as compared to control group (Table 3). In addition, the preantral population and the AFC significantly decreased to 0.22 fold and 0.33 fold respectively when compared with the control group. Conversely, the number of atretic follicles obtained from the irradiated ovaries was significantly higher than that obtained from the control group by about 136% (Table 3).

**Table 3:** Effect of Growth hormone (1mg/kg s.c.; once daily for 1 wk) on different classes of ovarian follicles and the mean diameter of antral follicles in rats subjected to single dose whole-body irradiation (3.2 Gy).

Treated groups	Primordial	Pre-antral	Antral	Atretic	The mean diameter of Antral follicles (μm)
**Control**	**26.33 ± 3.48**	**16.33 ± 3.18**	**8 ± 0.577**	**5.60 ± 1.20**	**272.89 ± 20.62**
**IR**	**4 ± 0.41** ^**a**^	**4.20 ± 0.71** ^**a**^	**2.33 ± 0.33** ^**a**^	**14.0 ± 0.91** ^**a**^	**182.02 ± 5.52** ^**a**^
**IR/GH**	**19.67 ± 2.90** ^**a,b**^	**15.00 ± 1.73** ^**b**^	**7.5 ± 0.64** ^**b**^	**3.40 ± 0.92** ^**b**^	**269.53 ±12.09** ^**b**^
**GH**	**20.66 ± 2.33** ^**a,b**^	**11.5 ± 1.55** ^**b**^	**7 ± 0.41** ^**b**^	**4.50 ± 0.95** ^**b**^	**267.39± 10.49** ^**b**^

-Data expressed as Mean ± SEM of at least three rats.

-a, b: Significantly different from control, radiation group, respectively at p <0.05 using one-way ANOVA followed by Tukey–Kramer as a post-hoc test.

Interestingly, the irradiated rats co-treated with GH showed higher numbers of ovarian follicles of different classes when compared to the irradiated group. However, they were still below control values in case of primordial cohort. However, in GH-treated group, primordial pool showed a significant decrease as compared to the control group, but no significant changes were found in the preantral and AFC populations. Besides the morphometric analysis of follicles, the mean diameter of antral follicle was assessed and results found that irradiation significantly reduced the mean diameter as compared to control group. However, both of irradiated and non-irradiated rats treated with GH showed a non-significant value of mean diameter of antral follicles when compared with the control group (Table 3).

**Fig 2:**
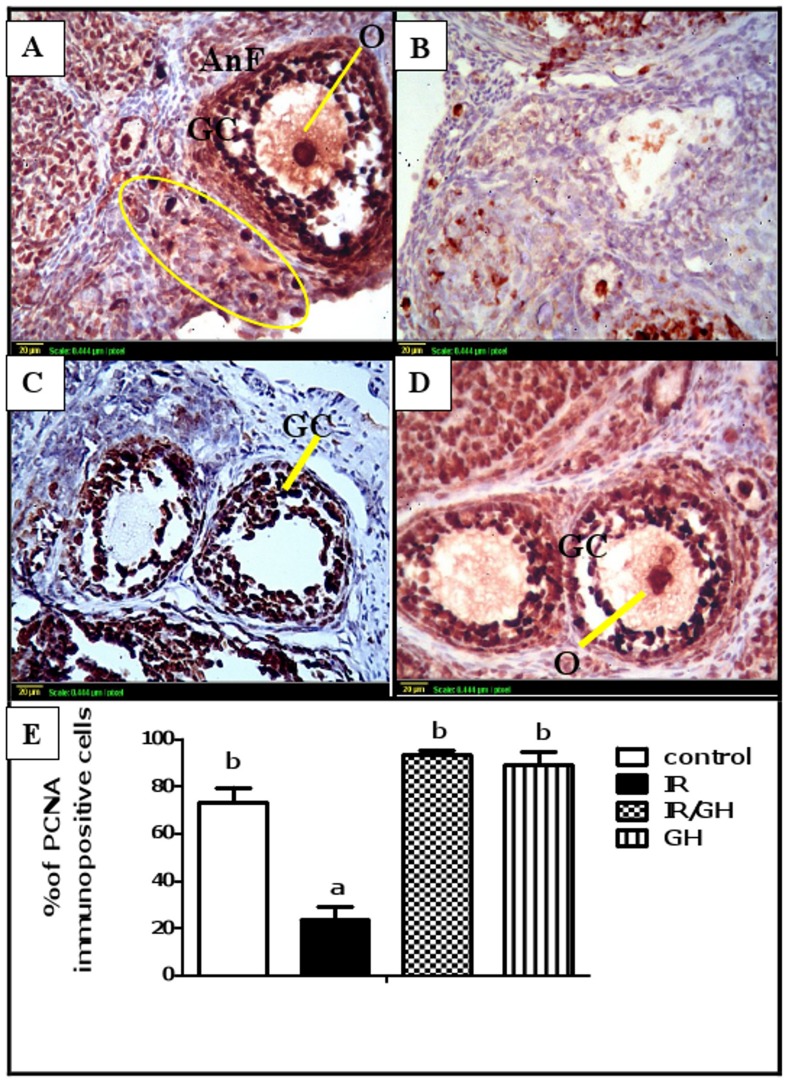
Follicular proliferation. Immunohistochemical localization of PCNA in ovarian follicles was studied 4 days after irradiation. **(A)**: Expression of PCNA in the control ovaries shows a large antral follicle (AnF) with positively stained (dark brown) granulosa cells (GC) and oocyte (O) and a cluster of primordial follicles with positively stained oocytes and stain-free pregranulosa cells around (oval). (**B)**: Expression of PCNA in ovaries of rats subjected to γ-irradiation (3.2 Gy) shows minimal expression. **(C) & (D)**: Expression of PCNA in ovaries of rats treated with GH 1 mg/kg for one week [either exposed to γ-radiation **(C)** or not **(D)]** shows a high PCNA expression of oocyte (O) and Granulosa cells (GC) (brown color). Scale bar, 20 μm. **(E)**: Quantitative Immunohistochemical staining of PCNA expressed as a percentage of immunopositive cells against the total number of granulosa cells across six high power fields (40×) from each rat section. Bars represent the Mean ± SEM of at least three independent experiments. a or b: Statistically significant from control or radiation group, respectively at P<0.05 using one-way ANOVA followed by Tukey–Kramer as a post-hoc test.

Moreover, proliferation of granulosa cells was assessed using immunohistochemical detection of the proliferation marker, PCNA. Results demonstrated that follicles at the same stage of development were at different states of maturation in control and irradiated ovaries. The control, GH-irradiated and GH-treated groups showed a strong nuclear immunostaining for PCNA in oocytes and proliferating granulosa cells of all growing follicles (Fig 2A, 2C & 2D). In addition, some primordial oocytes were positively stained with stain-free pregranulosa cells around.

In irradiated ovaries, the granulosa cells of almost follicles were mainly PCNA negative, and since a large number of follicles were atretic, they were also negative with degenerated oocytes inside. Additionally, only few late-antral follicles were resistant to irradiation and stained positive (Fig 2B). Moreover, our results were confirmed by quantitative PCNA expression. The percentage of immunopositive nuclei in irradiated rats significantly decreased as compared to control group (Fig 2E). However, no significant modifications were observed among groups treated with GH as compared with the control group.

### 3.4. Ovarian and uterine histology

**Fig 3:**
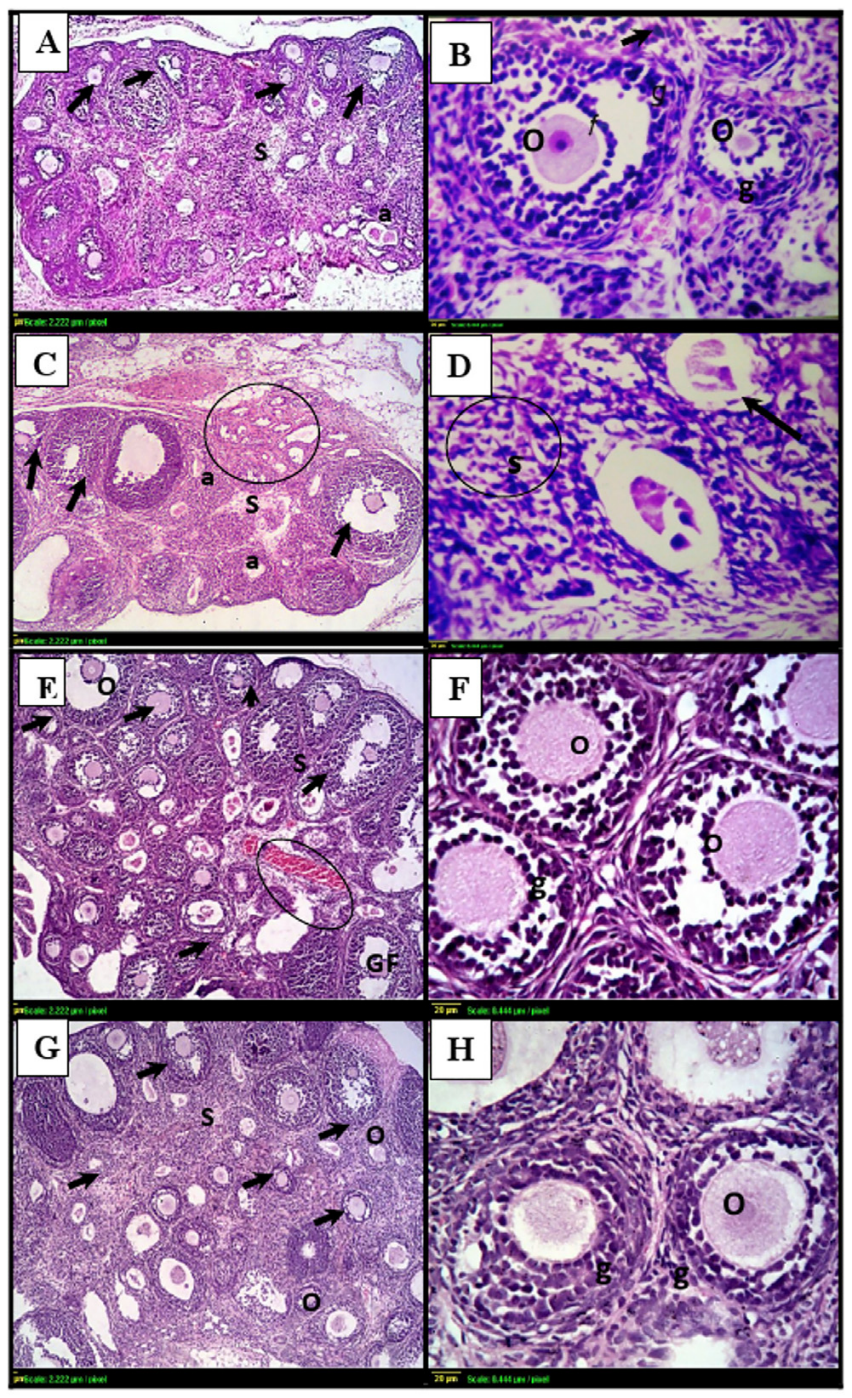
Photomicrographs of ovarian sections stained by hematoxylin and eosin. **(A)& (B)**: histological sections from control ovaries shows normal histopathological structure with multiple follicles of different stages (black arrows), intact oocytes (O) and granulosa cells (g). **(C)& (D)**: γ- irradiated ovarian sections shows few, if any, healthy follicles (arrows) with hemorrhage in the cortex (circle). Many small primary follicles are atretic (a) with degenerating oocytes and granulosa cells in irradiated ovaries. (**E)& (F):** GH-irradiated ovarian sections shows similar organization to the control group. **(G) & (H):** ovaries from animals treated with GH alone shows multiple growing follicles (arrows) with intact oocytes (O) and granulosa cells (g). Scale bar, 20 μm. gf: Graffian follicle, S: Stroma.

Ovarian sections of the control group showed normal histological structure of the cortex and medulla associated with multiple growing follicles in different stages (primordial, preantral and antral) and normal granulosa cell layers (Fig 3A & 3B). In addition, GH treatment alone didn’t modify the histological structure of ovary or growing follicles (Fig 3G & 3H). It was not the case in ovarian sections of irradiated group which showed many atretic follicles with degenerated oocytes and few growing ones, the predominant findings were the interstitial stromal cells and remnant of granulosa cells as shown in Fig 3C & 3D. On the other hand, GH ameliorated the radiation-induced ovarian follicular loss as shown in Fig 2E & 2F.

**Fig 4:**
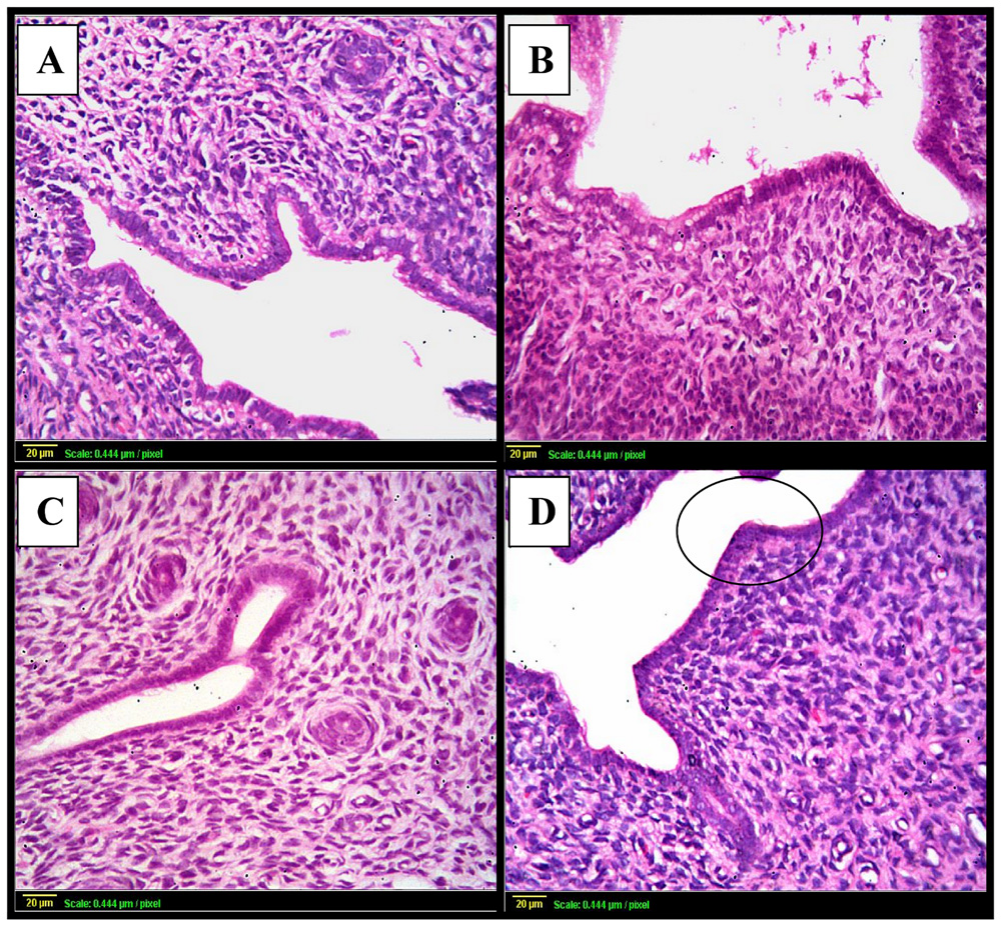
Photomicrographs of uterine sections stained by hematoxylin and eosin. **(A):** uterus from control rats shows normal histopathological structure of the mucosal lining epithelium (m) and the underlying lamina propria (p) with normal glandular structure (G). **(B):** uterine sections from rats subjected to γ- irradiation shows marked degeneration and stratification of the mucosal lining epithelium with multiple vacuoles (v) and thickening in the lamina propria **(C) & (D):** uterine sections taken from rats following GH treatment [either γ- irradiated **(C)** or not **(D)**] shows normal histological structure. Scale bar, 20 μm.

Besides, uterine sections from the control or GH-treated groups showed normal histopathological structure of the mucosal lining epithelium and the underlying lamina propria and normal glandular structure (Fig 4A, 4C & 4D). However, uterine sections from the irradiation group showed a marked degeneration and stratification of the mucosal lining epithelium with many vacuoles (Fig 4B).

### 3.5. Oxidative stress markers

Oxidative stress induced by radiation was assessed in rat ovaries by determination of GSH and lipid peroxides levels as well as GPx and GSHRx activities. As shown in Table 4, irradiation resulted in a marked GSH depletion to 19% and a significant decrease in GPx and GSHRx activities to 26.5% and 60%, respectively as compared to the control group. Additionally, irradiation induced a marked elevation in lipid peroxide levels as compared to control value. Interestingly, administration of GH significantly attenuated the irradiation-induced lipid peroxidation and improved the ovarian GSH content as well as the anti-oxidant enzymes activities. However, their values were still significantly different from the control group. Furthermore, GH alone showed no significant changes in GSH, lipid peroxides levels or GPx and GSHRx activities as compared to control values.

**Table 4:** Effect of Growth hormone (1mg/kg s.c.; once daily for 1 wk) on oxidative stress markers in rats subjected to single dose whole-body irradiation (3.2 Gy).

Treated groups	GSH (μmol/g wet tissue)	MDA(nmol/g wet tissue)	GPx (U/ mg protein)	GSHRx (mU/mg protein)
**Control**	2.64± 0.067 ^b^	9.94 ±0.570 ^b^	0.23±0.012 ^b^	1.78±0.082 ^b^
**IR**	0.51 ± 0.017 ^a^	38.90 ±1.20 ^a^	0.061±0.004 ^a^	1.07±0.043 ^a^
**IR/GH**	1.27 ±0.011 ^a,b^	26.02±1.02 ^a,b^	0.130± 0.012 ^a,b^	1.35±0.004 ^a,b^
**GH**	2.56 ± 0.140 ^b^	13.35± 1.36 ^b^	0.195 ±0.007 ^b^	1.69±0.033 ^b^

-Data are mean ± SEM (n = 6).

- a or b: Significantly different from control or radiation group, respectively at P <0.05 using one-way ANOVA followed by Tukey–Kramer as a post-hoc test

IR: irradiation; IR/GH: irradiation/Growth hormone; GH: Growth hormone; GSH, reduced glutathione; MDA, malondialdehyde; GPx, glutathione peroxidase; GSHRx, glutathione reductase.

### 3.6. Apoptotic markers

**Fig 5:**
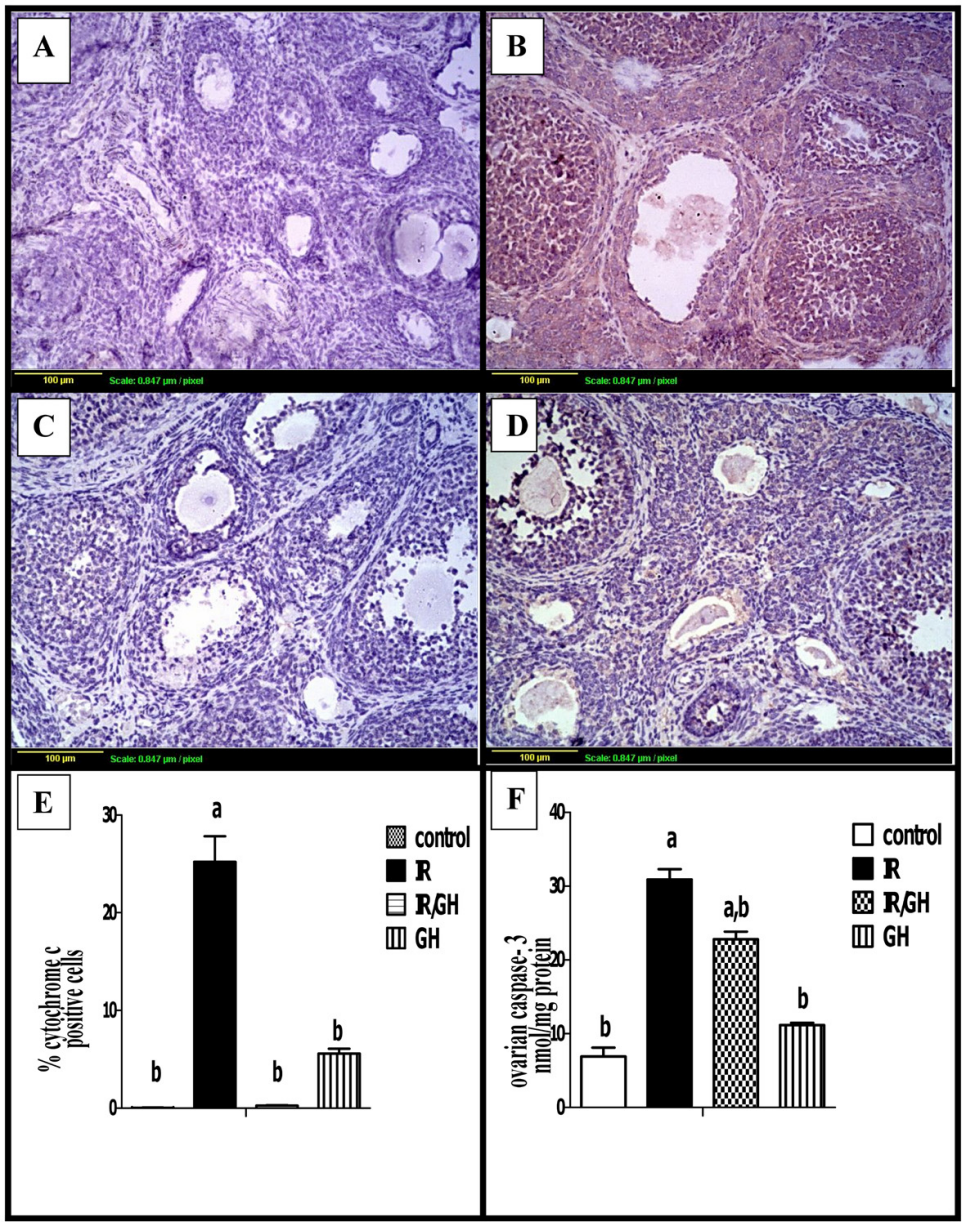
Immunohistochemical localization of cytochrome c. **(A):** section of ovary obtained from the control rats shows minimal degree of Cyto c expression (brown color). **(B):** section of ovary obtained from rats exposed to γ- irradiation (3.2 Gy) shows extensive Cyto c expression (brown color) in granulosa cells of all follicles. **(C):** section of ovary obtained from rats treated with GH (1mg/kg) and exposed to γ- irradiation (3.2 Gy) shows limited Cyto c expression (brow color). **(D):** section of ovary obtained from rats treated with GH (1mg/kg) alone for one week shows minimal Cyto c expression of granulosa cells (brown color). Scale bar, 20 μm. **(E):** Quantification of ovarian Cyto c staining represents the percentage of immunopositive cells to the total area of the microscopic high power field (20×); was averaged across 6 fields for each rat section of at least three independent experiments. **(F):** ovarian caspase-3 activity expressed as nmol/ mg protein. Each bar represents the Mean ± SEM for a group of 6 rats. a or b: Statistically significant from control or radiation group, respectively at P<0.05 using one way ANOVA followed by Tukey–Kramer as a post-hoc test.

The downstream Cyto c quantitative expression was assessed using immunohistochemical staining of ovarian sections. Results showed a minimal degree of Cyto c expression in granulosa cells of the control as well as the GH-treated group (Fig 5A & 5D). Radiation significantly increased the expression of Cyto c as shown by the intense brown staining of all growing follicles and a drastic increase in the percentage of immunopositive area as compared to control group (Fig 5B & 5E). Interestingly, concomitant treatment with GH significantly decreased the Cyto c expression and the percentages of positive areas were non-significant from the control group (Fig 5C & 5E).

In order to complete our assessment of irradiation-induced granulosa cell apoptosis, caspase-3 activity was also determined colorimetrically. Radiation caused a marked increase in ovarian caspase-3 activity by about 400% as compared with control group. In contrast, GH treatment decreased this elevation significantly by about 26% when compared with the irradiated group. Moreover, no significant change was observed in the group treated with GH alone as compared with the control value (Fig 5F).

### 3.7. IGF-1/IGF-1R axis assessment

**Fig 6:**
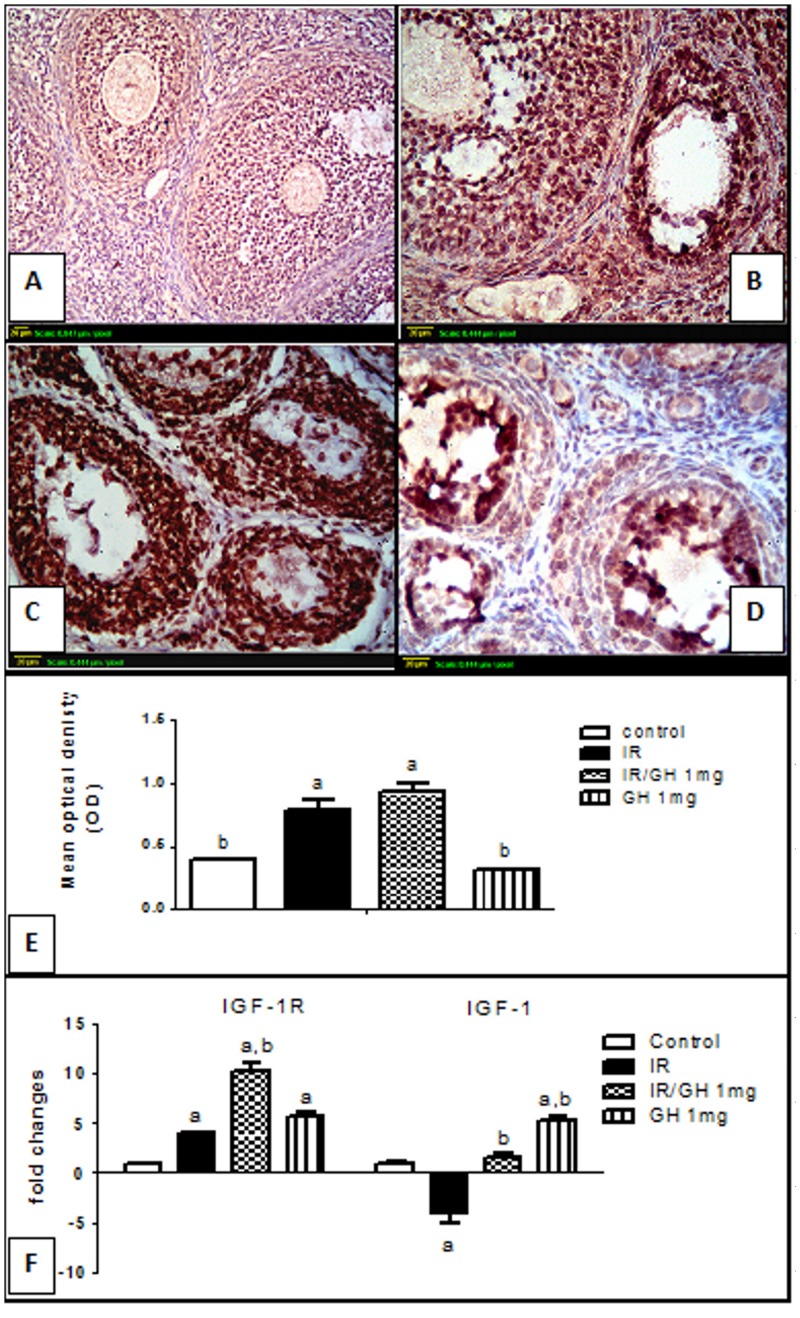
Immunohistochemical localization and Real-time quantitative Rt-PCR of insulin like growth factor-1 receptor (IGF-1R). **(A)**: section of ovary obtained from the control rats shows modest degree of IGF-1R expression (brown color). **(B)**: section of ovary obtained from rats subjected to γ- irradiation (3.2 Gy) shows extensive IGF-1R expression (brown color) in granulosa cells of almost all follicles. **(C)**: section of an ovary obtained from rats treated with GH and exposed to γ –irradiation (3.2 Gy) shows high IGF-1R expression (brown color). **(D)**: section of ovary obtained from rats treated with GH alone (1mg/kg; once daily for one week) shows modest IGF-1R expression similar to control ovaries (brown color).Scale bar, 20 μm. **(E)**: Semi-quantitative expression of ovarian IGF-1R staining represents the mean optical density of immunopositive cells of 6 high power fields (20×) of at least three independent experiments. **(F)**: Real-time quantitative RT-PCR of ovarian IGF-1 and IGF-1R genes expressed as fold changes relative to control group. Each bar represents the Mean ± SEM for a group of 3 rats. a or b: Statistically significant from control or radiation group, respectively at P<0.05 using one-way ANOVA followed by Tukey–Kramer as a post-hoc test.

The mRNA level of IGF-1 and IGF-1R were analyzed using Real time quantitative RT-PCR, the irradiated group showed a significant down-regulation for IGF-1 and up-regulation of IGF-1R by a factor of 4.15 and 4 respectively relative to the control group. Concurrent treatment with GH showed an up-regulation for both IGF-1R and IGF-1 genes by 10.3 and 2.2-fold respectively as compared with the control group. Furthermore, in GH alone treated group, both IGF-1 and IGF-1R genes were significantly up-regulated when compared with the control group (Fig 6F).

Beside genetic expression, serum and ovarian IGF-1 were determined four days post irradiation and the results showed that irradiation significantly decreased both serum and ovarian IGF-1 levels reaching 53.8% and 35% as compared with the control values (on average of 109.78± 3.63 ng/ml *vs*. 203.7± 3.4 ng/ml and 3.3± 0.18 ng/mg protein *vs* 9.82 ± 0.14 ng/mg protein in control respectively). This defect was significantly ameliorated by GH treatment. As was expected, GH alone showed a significant elevation in serum as well as ovarian IGF-1 to about 162% and 133% fold respectively as compared with the control group.

In addition, immunolocalization of IGF-1R was done and expressed as the mean OD of the immunopositive cells. The results demonstrated a modest degree of IGF-1R expression in ovaries of both the control and GH groups (Fig 6A & 6D), with no significant difference between them (Fig 6E). On the other hand, ovarian sections of the irradiated group showed an extensive IGF-1R expression in granulosa cells of most growing follicles (Fig 6B) with 1.9 fold increase in IGF-1R intensity as compared with the control values.

### 3.8. Mating with age related males

Estrous cycle was carried out one week prior to the beginning of the mating period. All rats showed normal cyclicity (4–5 day estrous cycle); however, most irradiated rats were arrested at the estrous and proestrous-estrous phase. After female rats became completely adult, the reproductive capacity in terms of fecundability and fecundity was examined. In the irradiated group, mating with age related males resulted in significant decline in female fertility than control ones. Only one of 10 irradiated females was pregnant as shown in Table 5.

**Table 5:** Reproductive performance of the control, irradiated and Growth hormone treated females.

Treated groups	Fecundability	Fecundity
Control	(10) 100%	8
IR	(1) 10%	3^a^
IR/GH	(10) 100%	9^b^
GH	(10) 100%	5^a,b^

Fecundability was expressed as a percentage of pregnant females among mated females (n = 10). Fecundity was expressed as the number of pups per mated females. Fecundity values represent the median; they were compared by Kruskal-Wallis’s test followed by Dunn’ multiple comparisons as a post-hoc test, and differences were considered significant when P,< 0.05. a or b: Significantly different from control or radiation group, respectively at P< 0.05.

In contrast, treatment of irradiated females with GH preserved their fertility and increased the ability of females to become pregnant to 100%. Additionally, no differences were found in gestation length or offspring morphologies among treatment groups.

## Discussion

New strategies are being developed to circumvent the adverse effects of the standard cytotoxic chemo-/ radiotherapy on ovarian function [45, 46]. Indeed, **Gómez-de-Segura and his coworkers** [22] have documented that giving hGH to irradiated rats promotes the adaptive process of the intestine and decreases the acute radiation-related negative effects, including mortality and weight loss. In addition, GH radioprotective effect was reported in the Chinese hamster ovary cells (CHO-4) that express the GHR, and this radioprotection was suggested to be due to DNA repairing mechanism [23]. In this line, no study has been documented on the GH potential radioprotective role in the female hypogonadism. With the hope to preserve the fertility of women undergoing radiotherapy, our study aimed to explore the molecular mechanisms underlying the potential radioprotective effect of GH on ovary. This was explored by studying different markers of oxidative stress, apoptosis, proliferation and growth factors in an experimental model of γ-irradiation-induced ovarian failure.

POF is manifested as hypergonadotropic hypogonadism, in this context; serum FSH and E2 levels have been used as markers of ovarian failure for decades [47]. Four days post-irradiation, serum E2 level was decreased, whereas FSH was drastically increased as compared with the control group, these results reflect the presence of a typical POF. On the other hand, administration of GH significantly improved the hormonal changes induced by irradiation. Our results were in accordance with those of previous studies, which suggested that GHRH plays a permissive role in amplification of ovarian FSH responsiveness probably via activation of the GH/IGF-1 axis [48, 49].

In addition, AMH was assessed in the different treatment groups because FSH and E2 measurements cannot express the ovarian reserve before infertility develops. AMH is considered to be the most sensitive marker for ovarian failure [50, 51] and represents the ovarian reserve more closely [52]. In the present study, undetectable levels of serum AMH and low AFC were found in female irradiated rats as compared with the control group. Interestingly, GH nearly normalized these defects in irradiated rats co-treated with GH for one week. These results were in accordance with those of human and experimental studies that previously reported undetectable levels of AMH following irradiation [53, 54]. High FSH levels detected following exposure to the gonadotoxic radiotherapy reflected the decrease in ovarian function and this was consistent with our low AMH levels. It is well known that AMH secretion is a gonadotropin-independent and its level is stable throughout menstrual cycles [55].

Moreover, it was reported that the healthy AFC serves as a marker of ovarian reserve and strongly correlates with the serum AMH [44]. Therefore, the morphometric analysis was carried out for different follicular populations. In the irradiated rats, a significant depletion of primordial follicle pool was detected as compared with the control group. Our results were in agreement with those of a previous study, which revealed that in the absence of AMH, primordial follicles are recruited at a faster rate and results in premature exhaustion of the primordial follicle pool [56]. Interestingly, treatment of irradiated rats with GH for one week improved the ovarian follicular populations of different classes when compared with the irradiated rats.

Indeed, GH acts on the recruitment of follicles proceeding to terminal growth and seems to be one of the cofactors necessary for survival and growth of follicles [57]. In this context, rats treated with GH alone showed a small but significant reduction in the primordial pool with no significant differences in the AFC as compared to the control group. Our results were in accordance with a previous study by **Slot et al**. [58], which reported that IGF-1 treatment of GHR/GHBP-KO mice for 14 days resulted in a reduced number of primordial follicles and an increased number of healthy antral follicles. They suggested that GH might play a role, either directly or indirectly, in the recruitment of primordial follicles into the growing pool. In addition, GH seems to protect antral follicles, directly or indirectly from undergoing atresia and to promote the follicular maturation to the preovulatory pool [59]. Collectively, GH regulates the effect of FSH on granulosa cells via increasing the synthesis of IGF-1. It also plays an essential role in ovarian function, including follicular development, estrogen synthesis and oocyte maturation [60, 61]. Our results provide *in vivo* evidence that GH itself is required for normal follicular development and ovulation rate.

In addition to morphometric analysis, the expression of the proliferation marker; PCNA was assessed. It is involved in many vital cellular processes including cell cycle regulation, apoptosis, cell survival and repair mechanisms [62]. In this study, the proliferation of granulosa cells four days post-irradiation markedly declined. This result may be explained by the fact that γ-irradiation activates p21, an inhibitor of cyclin-dependent kinases, through a p53-mediated mechanism [63, 64]. In addition, a study have showed that p21 is a selective negative regulator of PCNA-dependent DNA replication and nucleotide excision repair [65]. Nevertheless, expression of PCNA in granulosa cells begins upon the formation of a primary follicle and increases during the preovulatory follicular development under gonadotropin dependence [39]. In both control and GH-treated groups, expression of PCNA was detected in granulosa cells and oocytes and coincided with the initiation of follicle growth. PCNA immunoexpression was present only in the oocytes of most primordial follicles with stain-free pregranulosa cells. This suggests that PCNA is expressed by oocyte before the follicle is selected to grow. Our findings were in accordance with a previous study that suggested a role for this protein even in earlier stages of folliculogenesis [66]. Also, our results were in accordance with those of a previous study that found out that GH administration stimulated the ovarian cell proliferation accompanied with high PCNA immunoexpression. This is probably via the regulation of steroidogenesis and apoptosis processes [67].

In general, radiation-induced damaging effects occur via two pathways; direct and indirect. The direct one is through the ionization of DNA while the indirect way takes place by the production of reactive oxygen species (ROS) [68]. Consequently, many anti-oxidant enzymes and thiols (-SH) compete with the radiation- induced oxidative stress [69], reduce the transient free radicals and counteract these damaging effects [70]. In our study, ovarian GSH, as well as GPx and GSHRx activities were significantly depleted and lipid peroxides were drastically elevated following a single dose of γ-irradiation. However, GH administration for a week could improve the anti-oxidant levels when compared with the irradiated groups. In this context, a very recent study has reported that hGH administration showed a protective effect against ischemia/reperfusion-induced ovarian damage through this anti-oxidant mechanism [71]. Accordingly, our study proved that blocking oxidative stress and lipid peroxidation is another mechanism by which GH rescues the γ-irradiation-induced ovarian follicular loss.

In the light of the damaging effect of γ-irradiation, apoptosis is induced through ROS- mediated mechanisms [72]. ROS trigger the mitochondria-mediated apoptotic pathway and in turn, activate caspases cascade, particularly, caspase-3 [72, 73]. In general, follicular atresia or apoptosis being an essential component of ovarian function [74], occurs at all periods of life. It also can be activated by intrinsic or extrinsic pathways [75]. In the present study, Cyto c expression, as well as the executer caspase-3 activity was significantly increased in the irradiated group. Interestingly, GH ameliorated many changes in the downstream signaling pathways of apoptosis induced by irradiation. In agreement with these results, **Madrid & coworkers** [23] suggested that GH showed a dose- dependent protection against radiotherapy-induced cell death in CHO-4 cells due to DNA repairing mechanism. **Wu and coworkers** has recently reported that rhGH protects colorectal cancer cells from high dose irradiation by up-regulation of GADD45 and APEN protein expression, which is associated with cellular stress responses and DNA damage repair mechanisms [76]. Moreover, an *in vitro* data suggested that GH plays a role in follicular growth in the early gonadotropin-independent stage and could have a direct inhibitory action on follicle apoptosis [77]. So, another mechanism by which GH counteracts the radiation-induced ovarian damage may be through decreasing the follicular atresia mediated by apoptotic signals.

Furthermore, no study has explored the link between the GH radioprotective effect on ovary and IGF-1 axis. Indeed, the role of IGF-1 in stimulating and sustaining the granulosa cells proliferation is well documented [78]. IGF-1 and its receptor are positively expressed in the granulosa cells of healthy preantral and antral follicles [79]. Furthermore, IGF-1R activation decreases the ionizing radiation-induced cell death and initiates a cytoprotective signaling through a p38 pathway [80]. Accordingly, assessment of serum and ovarian IGF-1 levels along with ovarian expressions of IGF-1/IGF-1R were carried out in the different treatment groups. In the irradiated rats, serum and ovarian IGF-1 levels significantly dropped when compared with the control values. In contrast, real-time mRNA level and immunohistochemical localization of IGF-1R showed a small but significant up-regulation four days post irradiation. In contrast, it was reported that IGF-1R showed minimal immunostaining with increased follicular atresia 14 days post irradiation [54]. On the other hand, rats received GH for one week, either irradiated or not, showed a significant increase in ovarian IGF-1/IGF-1R on both transcription and translation level when compared with the control group. In this context, it was reported that GH action is mediated through enhancing the circulating and possibly the local IGF-1 [15]. Moreover, It may be relevant to note that IGF-1 activates the estrogen receptor and mediating its effects in the absence of E2 [81, 82]. Gamma-irradiation activates IGF-1R within 10 min triggering a cytoprotective signaling via DNA repair mechanism [80, 83]. Therefore, another mechanism for GH radioprotection in the ovary is possibly due to its potentiating effect on the IGF-1/IGF-1R axis, which mediates the cytoprotection and anti-oxidant effect.

In conclusion, the present study is the first one demonstrates that GH saves the ovarian reserve *in vivo* and provides evidence for ovarian radioprotection in the early stages of follicle maturation. The mechanisms by which GH achieves these effects may be through increasing hormonal secretion, improving ovarian cells proliferation and rescuing the primordial pool by ameliorating oxidative stress and apoptosis. In addition, the present study provides evidence for the first time that GH ameliorates the deleterious effects of irradiation via IGF-1-mediated processes. Since pharmacologic protection of the human resting primordial follicle pool during radiotherapy (and chemotherapy), so far has not been very successful, it will be very promising if GH can achieve such goal long-term.

However, this rat model only offers information on relative short-term effects of radiation, GH protection, and how these rat ovaries would look a few weeks later is still a question. Therefore, investigations of longer-term effects of GH on the primordial follicle pool after radiotherapy appear necessary before advancing into human trials. Further investigations are highly recommended to establish the clinical applicability of GH in cancer survivors with POF associated with radio-/chemo-therapy and to explore the possible interference with the anti-tumor activity of cytotoxic drugs.

## Abbreviations

AFC: antral follicle count
AMH: anti-Mullerian hormone
Cyto C: Cytochrome c
E2: 17β-estradiol
GH: Growth hormone
GPx: glutathione peroxidase
GSHRx: glutathione reductase
IGF-1R: IGF-1 receptor
PCNA: proliferating cell nuclear antigen
POF: premature ovarian failure
ROS: reactive oxygen species
TBS: Tris-buffered saline
